# A Novel Pix2Pix Enabled Traveling Wave-Based Fault Location Method

**DOI:** 10.3390/s21051633

**Published:** 2021-02-26

**Authors:** Jinxian Zhang, Qingwu Gong, Haojie Zhang, Yubo Wang, Yilin Wang

**Affiliations:** School of Electrical Engineering and Automation, Wuhan University, Wuhan 430072, Hubei, China; zhang2jx@whu.edu.cn (J.Z.); 2020102070019@whu.edu.cn (H.Z.); ybwang@whu.edu.cn (Y.W.); 2019282070164@whu.edu.cn (Y.W.)

**Keywords:** deep learning, Phasor Measurement Unit (PMU), Pix2Pix, YOLO v3, wavelet transform, fault location, traveling wave, location accuracy, transmission line

## Abstract

This paper proposes a new Image-to-Image Translation (Pix2Pix) enabled deep learning method for traveling wave-based fault location. Unlike the previous methods that require a high sampling frequency of the PMU, the proposed method can translate the scale 1 detail component image provided by the low frequency PMU data to higher frequency ones via the Pix2Pix. This allows us to significantly improve the fault location accuracy. Test results via the YOLO v3 object recognition algorithm show that the images generated by pix2pix can be accurately identified. This enables to improve the estimation accuracy of the arrival time of the traveling wave head, leading to better fault location outcomes.

## 1. Introduction

With the development of the smart grid, phasor measurement units (PMUs) play an increasingly important role in data-driven real-time fault location. PMUs have been successfully applied in high-voltage transmission systems [1]. The number of PMUs in North America has increased year by year since 2009 [2,3]. As wide area measurement devices, PMUs can integrate more signal acquisition modules, including the traveling wave acquisition module [4,5]. For real-time fault location, the sampling frequency of the traveling wave detection module in PMUs significantly affects fault location accuracy. Increasing the sampling frequency is positively correlated with improved accuracy for fault location. PMUs with higher sampling frequency can improve the visibility of power system dynamics, but also lead to a greater burden on effective data processing and higher cost of installations [2,6]. From an economic point of view, it is more feasible and effective to optimize the algorithm for processing data to improve the fault location accuracy instead of increasing PMU sampling speed.

Accurate fault location can save labor and material resources, reduce power outage time, and improve the safety of power grid operation. The traditional fault location methods mainly include impedance-based and traveling wave-based methods. The impedance-based methods use the post-fault steady fundamental voltage and current phasors to calculate the impedance of the fault circuit and estimate the fault distance [6,7,8,9]. Affected by the measured fault resistance, loading, and source parameters, the accuracy of fault location is not accurate enough. By contrast, traveling wave-based methods are not affected by system operation mode, transition resistance, fault type, and line distribution parameters [1,10,11,12]. The main idea is to calculate the time difference of the traveling wave between the terminal bus and the location of the fault. A new method of fault line selection and location based on D-PMU is proposed in [5]; through traveling wave signals collected by the D-PMU, fault locations are found accurately. However, this method requires clock synchronization and a high sampling frequency of PMUs [13,14].

Recently, advancements in the field of deep learning have aroused widespread attention. A dynamic Bayesian network (DBN) method to diagnose transient and intermittent faults is proposed in [15]. A real-time fault diagnosis methodology for complex systems is proposed in [16]. Object-oriented Bayesian networks are used to reduce complicated models, and Bayesian networks are used to solve the uncertainty problem of fault diagnosis. Deep learning also has wide application in fault location. The image feature extraction ability of a convolutional neural network (CNN) to extract the traveling wave head arrival time from multi-scale wavelet coefficients was utilized in [17]. Graph Convolutional Network (GCN) was used in [18] to extract the spatial features of a topology map for fault location in a distribution network. A CNN fault location method and an algorithm of joint PMU placement to improve location performance are proposed in [14]. However, the accuracy of the fault location is positively correlated with the accuracy of PMU sampling frequency. A fault detection method of YOLO v3 transmission line based on a convolutional block attention model is proposed in [19], solving the problem that the target to be detected in an aerial inspection image is easily affected by complex background and partial occlusion. Infrared thermal image recognition and application of power facilities based on a YOLO neural network is proposed in [20]. A novel method for detecting foreign objects on insulators based on the improved YOLO v3 is proposed in [21], and this method can be preliminarily aimed at unmanned aerial vehicle (UAV) inspections of transmission lines. The above-mentioned studies all show that YOLO has reached a wide range of applications in the electric power field because of its high accuracy. Since Pix2Pix was proposed, many outstanding achievements have been researched [22,23,24]. However, Pix2Pix has never been used in the research of power systems. The application of Pix2Pix in a power system needs to be researched.

There are many PMUs with insufficient sampling accuracy in the current high-voltage transmission system, and the maintenance of transmission lines often requires state grid workers to climb transmission towers for repairs. If those PMUs are not replaced, the fault location accuracy will not be accurate enough to locate a fault near one transmission tower, which will lead to a waste of human and material resources. If all of the PMUs are replaced, it will not only take a long time to install new ones in the corresponding transmission towers, but also cost a considerable amount. Therefore, an economical and efficient method to improve the accuracy of fault location needs to be studied. In this paper, a new method based on Pix2Pix is proposed to enhance the accuracy of traveling wave-based fault location. It mitigates the limitation that the accuracy of the fault location method is heavily dependent on the sampling frequency of PMUs. The PMU data are drawn into an image after wavelet transform, then, through the Pix2Pix algorithm, the image is transformed into a fake image that is similar to the image obtained by the higher-frequency PMU sampling. This allows us to achieve improved fault location accuracy.

The main contributions are summarized from two aspects: (1) An accurate fault location method based on Pix2Pix is proposed, which can significantly improve fault location accuracy. Moreover, unlike traditional fault location methods that require high sampling frequency of PMUs, the method can eliminate the dependence on the high sampling rate. (2) The YOLO v3 object recognition algorithm is applied in image quality evaluation, which replaces the tedious and repetitive work of manually verifying image quality and is more efficient. (3) Pix2Pix is proposed to locate fault points in transmission lines, and efficiently improve the accuracy of the arrival time of the traveling wave head. Improving the traveling wave head arrival time can improve the accuracy of fault location.

The remainder of this paper is organized as follows. The basic principles of the traveling wave-based fault location method, wavelet transform, Pix2Pix, and YOLO v3 are introduced in Section 2. In Section 3, the accuracy enhancement method for traveling wave fault location based on Pix2Pix is illustrated in detail. In Section 4, the training and test result of Pix2Pix is discussed, the results of YOLO v3 are evaluated, and the results of the accuracy enhancement method are proved. Finally, conclusions are presented in Section 5.

## 2. Principles

### 2.1. Traveling Wave-Based Fault Location Method

Traveling wave-based fault detection was proposed in the 1970s [25] as a concern of relay protection professionals. A protection technique based on the traveling wave was presented in the 1980s [26], and wavelet theory was used in fault location in the 1990s [27]. Since traveling wave-based fault location technology for transmission lines has the advantages of high ranging accuracy and wide application, it is now widely used in the protection of power grids. Depending on the existing PMUs between the two ends of the line, the fault location problem can be solved by the single-ended recording and double-ended synchronized recording methods.

**Figure 1:**
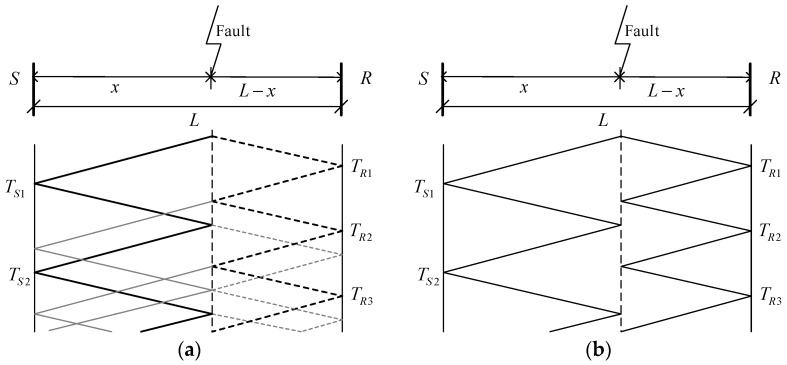
Lattice diagrams of (**a**) grounded fault and (**b**) ungrounded fault.

Figure 1 shows lattice diagrams illustrating the reflection and refraction of traveling waves initiated by grounded and ungrounded fault transients. The distance x from the fault point to bus S can be calculated by the single-ended and double-ended traveling wave fault location methods. $T_{S1}$ and $T_{R1}$ correspond to the times when the fault signal detail coefficients in scale 1 show their initial peaks for signals recorded at bus S and R, respectively. $T_{S2}$ corresponds to the time when the reflected backward traveling wave arrives at bus S. *v* is the velocity of propagation; it is shown in Equation (3), where L and C are the inductance and capacitance of the line per unit length. The difference between the two methods is the number of PMUs used. The single-ended method uses only one PMU and the double-ended method uses two PMUs, which are placed at both ends of the transmission line. The single-ended method uses $T_{S1}$ and $T_{S2}$ to locate the fault point, and double-ended uses $T_{S1}$ and $T_{R1}$. The formulas of the single-ended and double-ended methods are shown in Equations (1) and (2):

(1) $$\text{Single-ended}\;\;\;\;x=\frac{1}{2}v\Delta t=\frac{1}{2}v\left(T_{S2}-T_{S1}\right)$$

(2) $$\text{Double-ended}\;\;\;\;x=\frac{\left(T_{S1}-T_{R1}\right)v+L}{2}$$

(3) $$\mathrm{Velocity}\;\mathrm{of}\;\mathrm{propagation}\;\;\;\;v=\frac{1}{\sqrt{LC}}$$

The traditional fault location method can detect fault points accurately. The accuracy of the traveling wave head arrival time determines the accuracy of fault location. However, the arrival time highly depends on the high sampling frequency of the PMUs. Therefore, the sampling frequency of PMUs becomes the limitation in improving the accuracy of traditional fault location. In the next subsection, wavelet transform and multi-resolution analysis are introduced, which constitute an effective method to extract the arrival time of the fault-generated traveling wave head.

### 2.2. Wavelet Transform

A method named wavelet transform was applied in traveling wave fault detection in the 1970s [25,28,29]. It focuses on short intervals of high-frequency components and long intervals of low-frequency components and can improve the analysis of signals with local singularities [30]. The principle is that the wavelet transform has good detection ability for a transient traveling wave signal with a singularity that will appear when the transmission line fails. As the main tool for traveling wave detection and analysis, wavelet transform has been widely used in power system fault location [7]. The signal is transformed into detailed components with different scales through wavelet transform. Under the condition of low wavelet scale or high frequency band, the irregular detail component can replace the fault-generated traveling wave.

The current signals collected by PMUs are used for wavelet transform in this paper. Since the current signals are discrete signals, due to the orthogonality of the basis function, redundancy is eliminated to a certain extent, and the discretized time-frequency function has a stronger ability to reflect signal properties. Discrete wavelet transform (DWT) is shown in Equations (4) and (5), where *a* is the scale factor, *τ* is the translation factor, and *ψ* is the mother wavelet.

(4) $$DWTf(m,n)=\int_{R}f(t)\bar{\psi_{m,n}(t)}dt$$

(5) $$\psi_{m,n}(t)=a_{0}^{-\frac{m}{2}}\psi \left(\frac{t-n\tau_{0}a_{0}^{m}}{a_{0}^{m}}\right)$$

**Figure 2:**
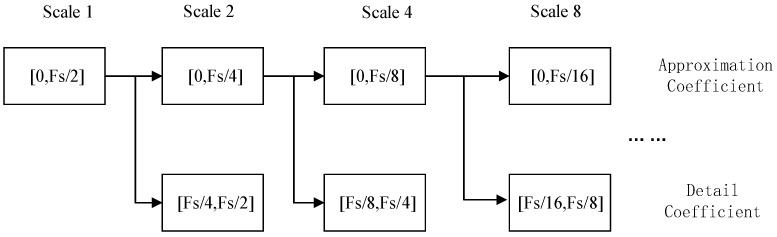
Tree structure of signal decomposition by discrete wavelet transform (DWT).

In this paper, multi-resolution analysis is selected as an effective algorithm to perform discrete wavelet transform. As shown in Figure 2, for a discrete signal f(n) under Fs sampling frequency, according to the Nyquist sampling theorem, it contains frequencies in the interval [0, Fs/2]. The DWT decomposes the signal f(n) into different scales to realize multi-scale analysis [31,32].

As indicated in [28], all singularities can be detected and characterized from the wavelet modulus maximum. It is proved in [30] that the local maximum modulus maximum of the detail component is the same as the singularity of the fault-generated traveling wave. The wave head of the fault-generated traveling wave can be obtained by finding the local modulus maximum of the detail component. It shows the feasibility of extracting the arrival time of the fault-generated traveling wave head by finding the first local modulus maxima of the detail component. This arrival time is most important for fault location, but its accuracy highly depends on the PMU sampling frequency. In the next subsection, we will introduce how Pix2Pix improves the accuracy of the fault-generated traveling wave head arrival time with low sampling frequency PMU.

### 2.3. Pix2Pix

The generative adversarial network (GAN) was proposed in 2014 [33]. GAN has a generator (G) and discriminator (D). G is used to generate samples, and D is used to judge whether the generated sample is a real sample. However, GAN generates images based on random noise z, and its output image is not controllable. In the later proposed conditional GAN (cGAN), an image is generated by random noise z and an input conditional variable [23]. Usually, each image-to-image translation problem uses a specific algorithm, and the essence of this algorithm is the mapping from pixel to pixel. Therefore, a general algorithm based on cGAN named Pix2Pix was proposed in [34] to solve this problem, which led to Pix2Pix being selected to improve the accuracy of fault location in this paper.

In the Pix2Pix network, the G uses U-Net, a network structure that is widely used in the field of image segmentation [35], to fully integrate features and improve details. The D uses Patch GAN to output a predicted probability value for each area (patch) of the input image, which is equivalent to evolving from judging whether the input is true or false to judging whether the input N × N area is true or false. As pointed out in [36], previous work found that the reason for adding L1 loss is because it can relatively reduce blurring; in addition, L1 loss also can make the images of the source domain and the target domain as close as possible. Since L1 can already guarantee the correctness of the low frequency, using Patch GAN can limit the high frequency in a reasonable expectation range. The Pix2Pix loss ($G^{*}$) is shown in Equations (6)–(8):

(6) $$L_{cGAN}(G,D)=E_{x,y}[\log D(x,y)]+E_{x,z}[\log(1-D(x,G(x,z))]$$

(7) $$L_{L1}(G)=E_{x,y,z}\left[\|y-G(x,z){\|}_{1}\right]$$

(8) $$G^{*}=\arg\min_{G}\max_{D}L_{cGAN}(G,D)+\lambda L_{L1}(G)$$

where x is the source domain image; y corresponds to the real image; z is the noise of input G; G(x,z) corresponds to the target domain image generated by G, which is based on the source domain image and random noise; D(x,y) corresponds to the probability that D judges whether the real image is real; and D(x,G(x,z)) is the probability that D judges whether the image generated by G is real.

**Figure 3:**
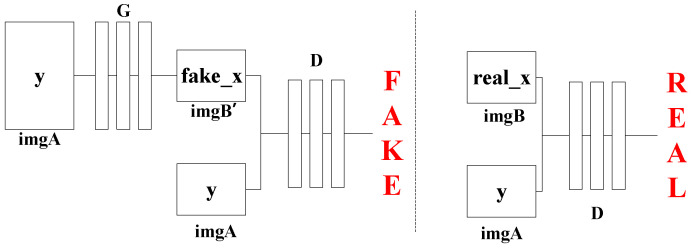
Pix2Pix training diagram.

A training diagram of Pix2Pix is shown in Figure 3. Generation network G has only one input, which is the condition y, which is an image named imgA, and the output is an image named imgB. After the training is completed, it can be transformed from imgA to imgB. The input of discriminating network D is x and y, where x is real imgB, which can be trained in pairs with imgA, so that the discriminator can judge whether imgB’ generated by G is real, and after the training is completed, it can correctly discriminate whether it is produced by G.

In this paper, Pix2Pix is used to translate the scale 1 detail component image provided by the low-frequency PMU data to a higher frequency. The scale 1 detail component images provided by the low-frequency and high-frequency PMU data are shown as imgA and imgB, respectively, in Figure 3. After translation by Pix2Pix, the location of the first local modulus maxima in the scale 1 detail component image becomes more accurate. This more accurate location can lead to a more accurate arrival time of the fault-generated traveling wave head, therefore, the accuracy of fault location with low-frequency PMU can be improved via Pix2Pix.

### 2.4. YOLO v3

YOLO was proposed by Joseph Redmon in 2015 as an object detection system based on a single neural network [37]. Its detection speed is much faster than similar algorithms, but as the first-generation algorithm, it has average ability to detect objects with overlapping edge features and often has problems with missing objects and generalization. YOLO v3 was proposed in 2018 to solve these problems. YOLO v3 adds a series of improvements based on the previous algorithm, mainly including FPN-like multi-scale prediction, multi-label classification, and a new residual neural network model, Darknet-53 [38]. These improvements enable YOLO v3 to overcome the shortcomings of previous versions. Currently, YOLO v3 is considered the ideal object detection algorithm while ensuring speed and high accuracy.

Compared with other object detection algorithms that usually use softmax classification, YOLO v3 uses logistic regression and binary cross-entropy loss to achieve classification, which ensures the algorithm’s multi-label tagging capability for the object. For object detection algorithms, the loss function is an important indicator to judge whether the model training has converged. The loss function of YOLO v3 (loss) is shown in Equation (9). The recall rate (R), precision (P), and mean average precision (mAP) can also be used to judge whether the model training has converged. The specific calculation is shown in Equations (9)–(12).

(9) $$\begin{array}{ll} loss= & \lambda_{coord}\sum_{i=0}^{S^{2}}\sum_{j=0}^{B}\prod {}_{ij}^{obj}[{\left(x_{i}-\hat{x_{i}}\right)}^{2}+{\left(y_{i}-\hat{y_{i}}\right)}^{2}]\\ & +\lambda_{coord}\sum_{i=0}^{S^{2}}\sum_{j=0}^{B}\prod {}_{ij}^{obj}[{\left(\sqrt{\omega_{i}}-\sqrt{\hat{w_{i}}}\right)}^{2}+{\left(\sqrt{h_{i}}-\sqrt{\hat{h_{i}}}\right)}^{2}]\\ & +\sum_{i=0}^{S^{2}}\sum_{j=0}^{B}\prod {}_{ij}^{obj}[{\left(C_{i}-\hat{C_{i}}\right)}^{2}+\lambda_{noobj}\sum_{i=0}^{S^{2}}\sum_{j=0}^{B}\prod {}_{ij}^{obj}[{\left(C_{i}-\hat{C_{i}}\right)}^{2}\\ & +\sum_{i=0}^{S^{2}}\prod {}_{i}^{obj}\sum_{c\in classes}{\left(p_{i}(c)-\hat{p_{i}}(c)\right)}^{2} \end{array}$$

where $x,\underset{}{}y,\underset{}{}\omega ,\underset{}{}C,\underset{}{}p$ are the network prediction values of the model output; $\hat{x},\hat{y},\hat{\omega },\hat{C},\hat{p}$ are the label values; $\prod_{i}^{obj}$ refers to the object is in the *i*th grid cell; $\prod_{ij}^{obj}$ refers to the object in the *j*th prediction bounding box of the *i*th grid cell; and $\prod_{ij}^{noobj}$ refers to the object not in the *j*th prediction bounding box of the *i*th grid cell.

(10) $$\mathrm{Precision}:\;\;\;\;P=\frac{T_{P}}{F_{P}+T_{P}}$$

(11) $$\mathrm{Recall}\;\mathrm{rate}:\;\;\;\;R=\frac{T_{P}}{T_{P}+F_{N}}$$

(12) $$\mathrm{mAP}:\;\;\;\;mAP=\frac{\sum_{n=1}^{N}\frac{1}{11}\sum_{R\in \left\{0,0.1,\ldots ,1\right\}}P_{interp}(R)}{N}$$

where *T_P_* (true positive) refers to the number of positive samples that are correctly classified; *F_P_* (false positive) refers to the number of negative samples that are judged as positive samples; *F_N_* (false negative) refers to the number of positive samples that are judged as negative samples; *T_N_* (true negative) refers to the number of negative samples that are judged as negative samples; and $P_{interp}(R)$ is the maximum precision value when *R* reaches its maximum value. The precision rate is defined as the proportion of real examples in all images identified as positive examples, and the recall rate is defined as the ratio of real examples to all positive examples in the sample. It is often difficult to balance between precision and recall rate, and an excellent object detection algorithm often needs to maintain high precision while increasing the recall rate. mAP is the average value of AP. For object detection algorithms, detection is often oriented to multiple objects in multiple categories, and the average AP values of different categories often have different sizes, therefore mAP is introduced as a comprehensive evaluation indicator of the object detection algorithm. When AP or mAP is calculated, the intersection value over union (IOU) is always set to 0.5.

In this paper, the indicators recall rate, precision, and mean average precision and loss are also used to judge whether the training is completed, and the output of YOLO v3 is a supplementary indicator used to evaluate the status of Pix2Pix training and the quality of images generated by Pix2Pix. More details are discussed in Section 3.3.

## 3. Accuracy Improvement Method

### 3.1. Dataset Generation

Regarding the precise requirements for fault location, it is generally required that the fault location error of transmission line should not exceed 300–400 m, which is the distance between two transmission towers. Based on the velocity of traveling wave propagation v in the transmission line being close to the speed of the light, its fault location error should be within 300 m. Therefore, the sampling frequency should bigger than 1 MHz. In this paper, we chose 1 and 2 MHz as the sampling frequencies of PMUs to build a simulation system.

**Figure 4:**
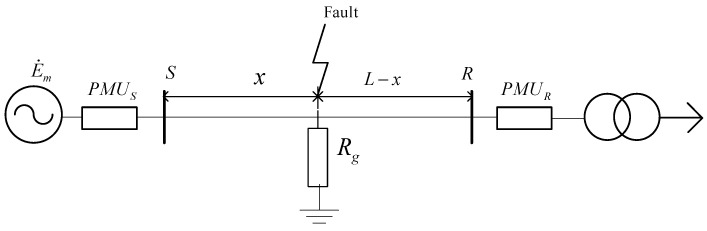
Equivalent network diagram.

Pix2Pix training requires a large number of paired images. To obtain 1 and 2 MHz images, Simulink was used to build a simple power grid model, and PMUs were placed at both ends of the transmission line to obtain traveling wave data. The equivalent circuit diagram is shown in Figure 4.

**Figure 5:**
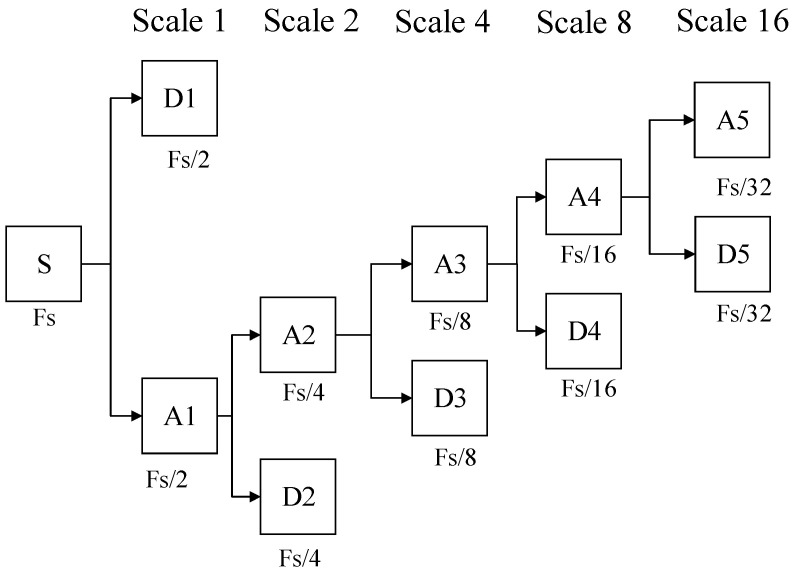
Five-layer multi-resolution analysis structure.

The five-layer multi-resolution analysis tree structure is shown in Figure 5, where *s* corresponds to the original signal; *a* corresponds to the low-frequency part, which is obtained by the ideal low-pass filter; and *D* is the high-frequency part, which is obtained by the ideal high-pass filter. Multi-resolution analysis gradually improves the frequency resolution by decomposing the low-frequency space. It can separate the signal into high- and low-frequency components through high- and low-pass filters. The decomposed results of the signal at different scales are linked together to reconstruct the original signal [39,40]. The fault-generated traveling wave can be decomposed into approximate and detailed components. The original signal *s* can be obtained by reconstructing and superimposing the frequency bands of a5, d1, d2, d3, d4, and d5, which can be expressed as Equation (13):

(13) $$s=a_{5}+d_{1}+\;d_{2}+\;d_{3}+d_{4}+\;d_{5}$$

In this paper, the traveling wave fault signal is decomposed into different frequency bands by DWT to achieve multi-resolution analysis [26,41]. The fault-generated traveling wave signal is transformed by a 3 dB wavelet, and the scale 1 detail component image is intercepted in seven sampling intervals before and after the fault occurrence time. The image is cropped to a size of 256 × 256, then the processed image is used as the conditional input of the Pix2Pix network to generate an image with higher sampling frequency.

### 3.2. Pix2Pix Training

In the training process, the goal of G is to generate as many images as possible to discriminator D, and the goal of D is to separate as many of the images generated by G from the real images as possible. Since the generator is initialized randomly at the beginning of training, it makes the corresponding data distribution far away from the real data distribution and D can easily distinguish the generated and real images. However, with increased training epochs, the images generated by G get closer and closer to the actual data distribution, and it becomes difficult for D to distinguish them. Usually, D loss decreases during training, while GAN loss oscillates. The loss curve does not usually reveal much information, so it is hard to judge whether the training is successful or not just based on the loss curve. Therefore, additional evaluation metrics are needed to evaluate the quality of the generated images.

### 3.3. Evaluation Metrics

Quality evaluation of the generated images is an open problem [34]. Although direct visual inspection of samples is a common and intuitive method to evaluate GAN, evaluating the quality of generated images by human vision is biased and difficult to reproduce, and cannot fully reflect the capabilities of the model [42]. YOLO v3 is an object detection algorithm that belongs to the one-stage series. It regards object detection as a type of regression problem and can directly predict the coordinates and class probability of the bounding box of the object from the input image. Therefore, its detection speed is very fast, and real-time object detection can be achieved with high accuracy [38].

In this paper, YOLO v3 is used to recognize Pix2Pix trained images, and the output of YOLO v3 is used to judge the training status of Pix2Pix and evaluate the quality of images generated by G. In the training process, indicators such as recall rate, precision and mean average precision, and loss can also be used to judge whether the training is completed. By training a large number of real pictures generated by Simulink, YOLO v3 can recognize the scale 1 detail component images generated by G. The output of YOLO v3 is used to evaluate the status of Pix2Pix training and the quality of images generated by Pix2Pix. The output of YOLO v3 is always an image with a bounding box and prediction value. The higher the prediction value, the higher the image quality.

### 3.4. Accuracy Improvement Effect Evaluation

The improved accuracy of the arrival time of the fault-generated traveling wave head is mainly reflected in the difference in its value between 1 and 2 MHz sampling frequency. In the simulation process, the fault-generated traveling wave head arrival time is the same as the time corresponding to the location of the first local modulus maximum.

The arrival time under 1 MHz sampling frequency is taken as a reference, and it is compared with the arrival time under 2 MHz sampling frequency and that obtained from the image generated by Pix2Pix. The formulas for arrival time error $\varepsilon_{1}$ and $\varepsilon_{fake}$ are shown in Equations (14) and (15), and the percentage of accuracy improvement effect $\eta_{fake}$ is shown in Equation (16):

(14) $$\varepsilon_{1}=\frac{t_{2}-t_{1}}{t_{2}}\times 100\%$$

(15) $$\varepsilon_{fake}=\frac{t_{fake}-t_{2}}{t_{2}}\times 100\%$$

(16) $$\eta_{fake}=\frac{\varepsilon_{1}-\varepsilon_{fake}}{\varepsilon_{1}}\times 100\%$$

where $t_{1}$ and $t_{2}$ represent the arrival time of the fault-generated traveling wave head obtained by the simulation under 1 and 2 MHz sampling frequency, respectively, and $t_{fake}$ is the arrival time obtained from the image generated by Pix2Pix.

The evaluation model is also built from the equivalent circuit diagram shown in Figure 4. The accuracy enhancement effect of fault location can be evaluated by calculating the distance between the fault point and bus S; in this paper, that distance is set as a fixed value, $x_{true}$. $T_{S1}$, $T_{S2}$, and $T_{R1}$ can be sampled by PMUs from Simulink under 1 MHz sampling frequency and different fault types. The measured distance $x_{location}$ can be calculated by different recording methods according to Equations (1) and (2). Moreover, the Pix2Pix-based fault location algorithm is also based on double-ended recording. The formula for distance x from the fault point to bus S is shown in Equations (17) and (18), where $t_{fake}$ corresponds to the arrival time of the fault traveling wave from the image generated by Pix2Pix and the image containing $T_{S1}$ is used as the input of Pix2Pix.

(17) $$\text{Single-ended}\;\mathrm{recording}:\;\;\;\;x=\frac{1}{2}v\Delta t=\frac{1}{2}v\left(T_{S2}-t_{fake}\right)$$

(18) $$\text{Double-ended}\;\mathrm{recording}:\;\;\;\;x=\frac{\left(t_{fake}-T_{R1}\right)v+L}{2}$$

Single phase-to-ground fault and phase-to-phase fault are selected to test the effect of accuracy enhancement. The improved accuracy of fault location can be determined by $\varepsilon_{location}$, which is shown in Equation (19), corresponding to the error between the true fault point and the fault point after measurement and calculation.

(19) $$\varepsilon_{location}=\frac{x_{location}-x_{true}}{x_{true}}\times 100\%$$

### 3.5. Flowchart of Accuracy Improvement Method

**Figure 6:**
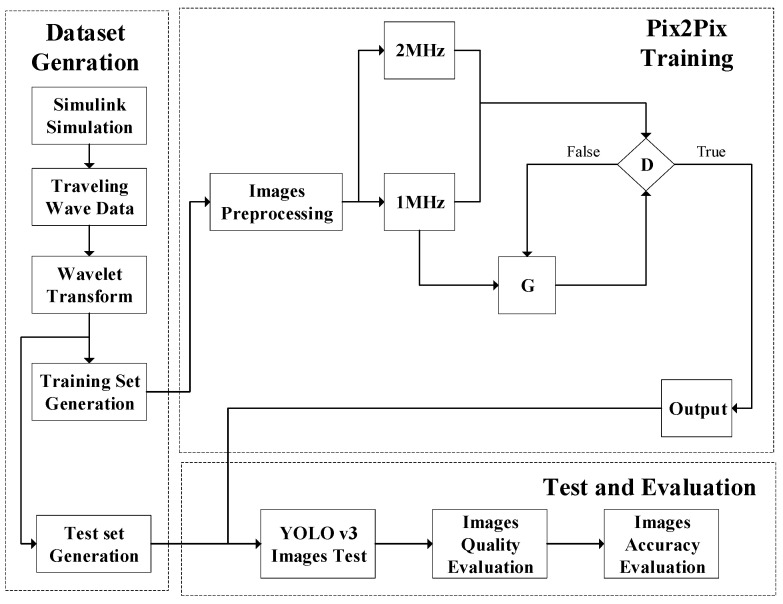
Flowchart of accuracy improvement method.

A flowchart of the accuracy improvement method is shown in Figure 6 to provide a clear overview. First, the current sampling data are obtained by the Simulink simulation, and then, through wavelet transform, the scale 1 detail component images are generated and preprocessed to obtain the training and test sets. The training set is used to train Pix2Pix, and the test set is used to test and evaluate the effect of Pix2Pix-generated images. After the training process, YOLO v3 is used to evaluate the quality of images generated by G, then the images in the test set are used to test the accuracy improvement effect.

## 4. Analysis of Results

### 4.1. Simulation Results

The hardware used for simulation was Intel(R) Xeon(R) CPU E5-2620 @2.10 GHz × 32, 64 GB memory, with GeForce GTX 1080Ti GPU. The operating system was Ubuntu 16.0.4, and the Python version was 3.6. The Pix2Pix model was based on the PyTorch deep learning framework. The simulation model was built according to the equivalent circuit diagram shown in Figure 4.

Through Simulink, phase-to-phase faults and ground faults were simulated, the system voltage level was set to 110 kV, and the total length of the transmission line was 50 km. Due to the need to obtain traveling wave data, the transmission line adopted a distributed parameter model, the positive sequence resistance was set to 0.01273 Ohms/km, the positive sequence inductance was 0.9337 mH/km, and the positive sequence capacitance was 0.01274 µF/km. Therefore, the velocity of propagation *v* is 289,942.31 km/s. The fault occurrence time was generated by random numbers and lasted for 0.3 s.

**Figure 7:**
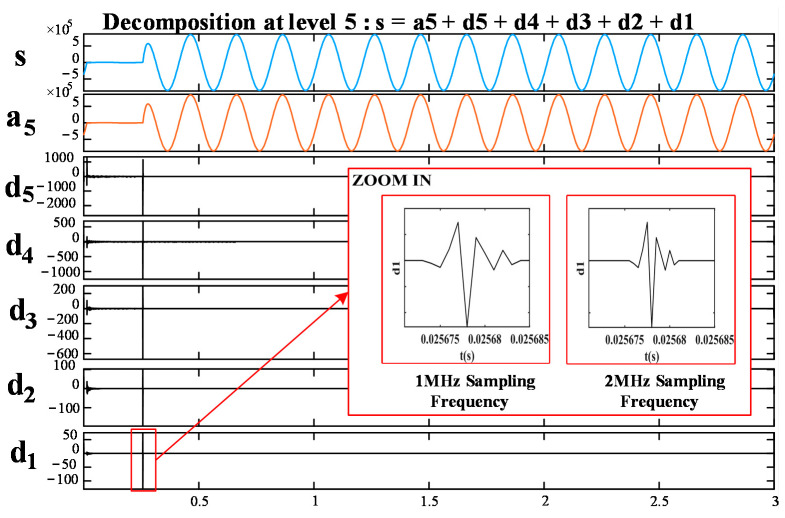
Wavelet decomposition structure.

Moreover, under the same fault occurrence time, 2 and 1 MHz sampling frequencies were used to sample the current of the faulted circuit, and the current data were obtained. According to Figure 4, the fault data were transformed by a 3 dB wavelet, and the approximate and detail components are shown in Figure 7. The detailed component image is enlarged in scale 1 to 5 sampling intervals before and after the fault occurs. Figure 7 shows zoomed-in scale 1 detail component images with 1 and 2 MHz sampling frequencies from d1. Then the two images are combined into one image. According to the above method, 2000 images were generated in batches, and 80% were used for training and 20% for testing.

### 4.2. Pix2Pix Training Result

The size of the scale 1 detail component image was set to 256 × 256, the discriminator patch size was set to 70 × 70, the weight of the feature matching loss function *λ* was set to 100, the batch size was set to 1, the learning rate was set to 0.0002, and the momentum parameters were β1=0.5,β2=0.999.

**Figure 8:**
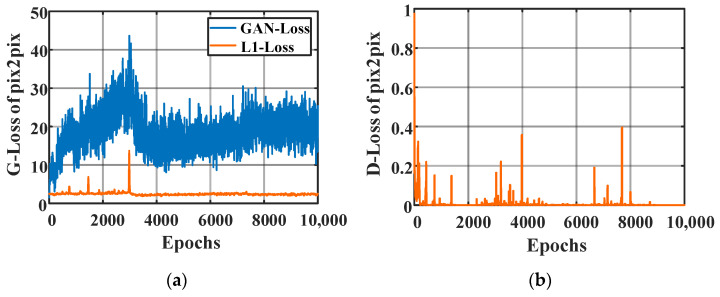
Loss of Pix2Pix: (**a**) loss of generator; (**b**) loss of discriminator.

In this paper, a scale 1 detail component image was transformed from 1 to 2 MHz sampling frequency through Pix2Pix. The standard for judging the accuracy of images generated by Pix2Pix should be that the arrival time of the fault-generated traveling wave head in the fake image is within the time interval [$t_{1},t_{2}$]. As shown in Figure 8, this corresponds to the loss of Pix2Pix, with the loss of the generator (Figure 8a) and the discriminator (Figure 8b).

**Figure 9:**
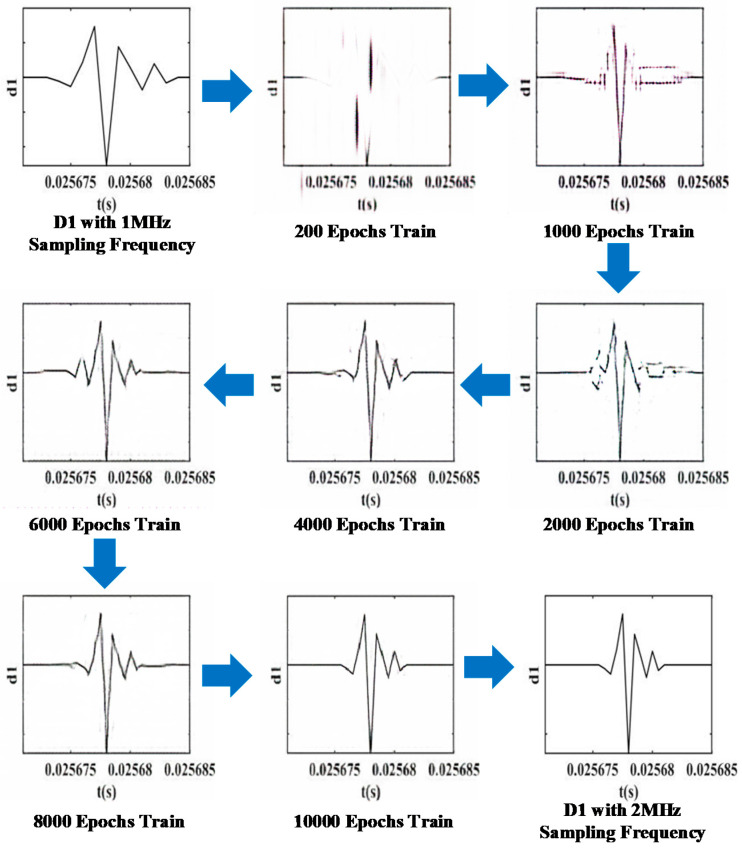
Pix2Pix training process.

According to the changes in the distribution of the generated data during the Pix2Pix training process (Figure 9), it can be found that the randomly generated data and the actual data distribution are different at the beginning of training. However, as the training epochs increase, the difference between the two distributions becomes smaller.

Figure 9 shows the training images generated by Pix2Pix in epochs 200, 1000, 2000, 4000, 6000, 8000, and 10,000. The results show that the data distribution generated by Pix2Pix gets closer and closer to the real data distribution.

### 4.3. Image Quality Evaluation

**Figure 10:**
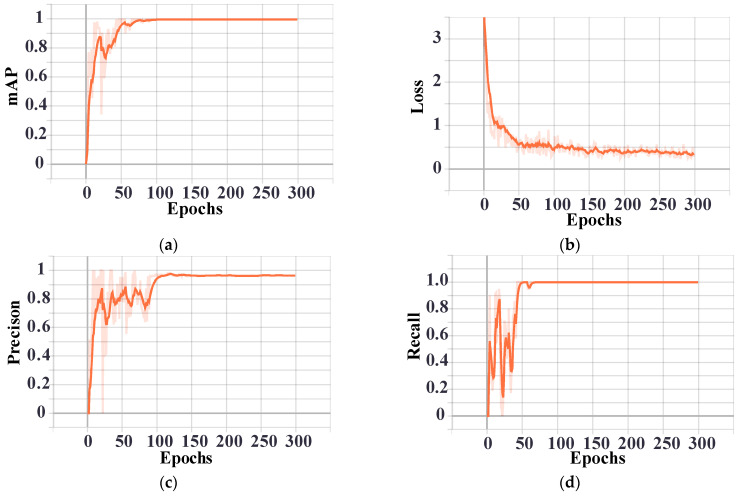
(**a**) mAP, (**b**) loss, (**c**) precision, and (**d**) recall of YOLO v3.

In this paper, the images generated by Pix2Pix were recognized by YOLO v3. The training set consists of 200 standard traveling wave head images generated by Simulink simulation at a 2 MHz sampling frequency, with 80% of images used for training and 20% for testing. The batch size was set to 8 and the epochs of training was set to 300. Recognition results such as precision, recall rate, loss, and mAP are shown in Figure 10. In order to facilitate observation of the changing trend, the orange curve shown in Figure 10 represents the changing trend of the training, smoothed by the default settings of the TensorBoard. The shade of the orange curve represents its actual value in each epoch. The results show that as the number of epochs increases, the recognition rate will increase. However, there is a time when the training epochs reach a particular value, the recognition rate will not change significantly, and, finally, the detail component image can be accurately recognized. It can be seen from Figure 10 that YOLO training has been completed.

A summary of the test result is shown in Table 1. The precision (P) is 98.1%, the recall rate (R) is 100%, the mean average precision under the IOU = 0.5 is 99.5%, and the average recognition time is 280.4 ms per image with a batch size of 8.

**Figure 11:**
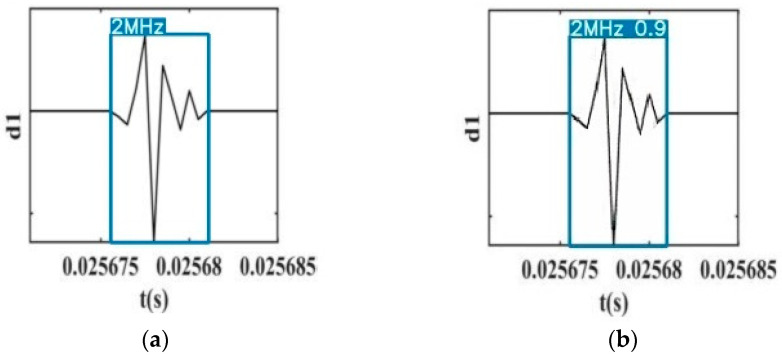
G-generated image detection results of YOLO v3: (**a**) test result; (**b**) detection result.

In this paper, 30 images generated by G were not in the training or test dataset, and all of them were detected by YOLO v3. The detection result shows that all of those images were successfully recognized by YOLO v3. Figure 11 shows the output of YOLO v3. The test result is shown in Figure 11a and the detection result in Figure 11b. From the detection result, the scale 1 detail component image under 2 MHz sampling frequency is detected, and its prediction value is 0.9, which means the training of Pix2Pix is completed and the quality of G-generated images is high. Therefore, the process of accuracy improvement can be tested.

### 4.4. Result of Accuracy Improvement

According to the definitions of Equation (16), we will show the accuracy improvement effect of some samples in the test sample set. Table 2 shows the results of the arrival time of the fault-generated traveling wave head obtained by Pix2Pix training on 1 and 2 MHz detail component image pairs. The percentage values of accuracy improvement in the multiple tests are also shown in Table 2. The accuracy of the arrival time of the fault-generated traveling wave head corresponding to each image improved in different degrees, and the percentage of accuracy improvement is 65.19% on average. It can be seen that the data distribution of the 2 MHz traveling wave image generated by Pix2Pix can accurately approximate the actual data distribution and generate a scale 1 detail component image under a sampling frequency close to 2 MHz, which can effectively improve the accuracy of fault location.

The results of changing the different types of faults and the locations of fault points are shown in Table 3. When phase-to-phase fault occurs in the transmission line, the average error of single-ended fault location is 0.71 km, double-ended traveling wave fault location is 0.306 km, and double-ended traveling wave fault location based on Pix2Pix is 0.184 km. When a ground fault occurs in the transmission line, the average error of single-ended fault location is 0.686 km, double-ended traveling wave fault location is 0.352 km, and double-ended traveling wave fault location based on Pix2Pix is 0.25 km. The results illustrate that the location error of the double-ended method based on Pix2Pix is lower than that of the traditional fault location methods. The accuracy of the fault location is obviously improved by Pix2Pix, even under different types of faults, so the Pix2Pix-based method can also effectively improve the accuracy of fault location.

## 5. Conclusions

In this paper, a method for enhancing the accuracy of traveling wave fault location based on Pix2Pix is proposed. Through the training and testing of data generated by a Simulink simulation, YOLO v3 image recognition, and evaluation of the generated image quality, and based on the accuracy improvement performance test, the method’s effectiveness is proved.

The simulation results show that the Pix2Pix-based accuracy enhancement method solves the limitation that fault location accuracy depends on the PMU sampling frequency. Through Pix2Pix, images obtained from original PMU sampling data can be translated to images obtained with higher sampling frequency. The average accuracy improvement is 65.19%, which means the Pix2Pix-based traveling wave fault location accuracy enhancement method can effectively reduce the fault location range and save labor and material resources. Moreover, it also can improve the safety and stability of power system operation. In addition, this method does not require the installation of additional measuring equipment, and it can save on a lot of equipment update and installation costs.

Current data are the only required data in this method. At present, this method aims at improving fault location accuracy for traveling wave-based methods, and the output image resolution is 256 × 256. In the future, we will focus on higher-resolution image processing to achieve better accuracy improvement.

## Figures and Tables

**Table 1:** Summary of YOLO v3 test result. P, precision; R, recall; mAP, mean average precision.

Model	Class	P	R	mAP@0.5	Average Test Time
YOLO v3	2 MHz	98.1%	100%	99.5%	280.4 ms

**Table 2:** Arrival time of traveling wave head and accuracy improvement effect.

No.	$t_{fake}/s$	$t_{1}/s$	$t_{2}/s$	$\eta_{fake}$
1	0.0451842	0.0451830	0.0451855	48.00%
2	0.1469605	0.1469589	0.1469608	84.21%
3	0.1066807	0.1066789	0.1066809	90.00%
4	0.0478147	0.0478128	0.0478154	73.08%
5	0.0273322	0.0273310	0.0273327	70.59%
6	0.0667762	0.0667750	0.0667775	48.00%
7	0.0620182	0.0620169	0.0620196	48.15%
8	0.1018685	0.1018670	0.1018687	88.24%
9	0.0135866	0.0135865	0.0135866	94.44%
10	0.0270906	0.0270889	0.0270916	62.96%
11	0.0449620	0.0449500	0.0449750	48.00%
12	0.0531665	0.0531650	0.0531666	93.75%
13	0.0632626	0.0632610	0.0632626	100.00%
14	0.0647566	0.0647550	0.0647567	94.12%
15	0.0936843	0.0936830	0.0936846	81.25%
16	0.0653522	0.0653510	0.0653535	48.00%
17	0.1066807	0.1066788	0.1066808	95.00%
18	0.0867760	0.0867750	0.0867775	40.00%
19	0.1081824	0.1081810	0.1081828	77.78%
20	0.1069024	0.1069009	0.1069026	88.24%
21	0.1226621	0.1226610	0.1226623	84.62%
22	0.0141324	0.0141305	0.0141326	90.48%
23	0.1043904	0.1043889	0.1043904	100.00%
24	0.0624564	0.0624548	0.0624576	57.14%
25	0.1025027	0.1025010	0.1025027	100.00%
26	0.1484780	0.1484767	0.1484793	50.00%
27	0.1044980	0.1044969	0.1044986	64.71%
28	0.0125204	0.0125190	0.0125206	87.50%
29	0.0359905	0.0359888	0.0359914	65.38%
30	0.1417161	0.1417148	0.1417165	76.47%
Average percentage of accuracy improvement $\eta_{fake}$.				65.19%

**Table 3:** Contrast of simulation results.

Fault Location $x_{true}$	Fault Type	Recording Method	Fault Point $x_{location}$	Absolute Error	Relative Error
10 km	Ag	Single-ended	10.19 km	0.19 km	1.9%
		Double-ended	10.17 km	0.17 km	1.7%
		Double-ended based on Pix2Pix	10.08 km	0.08 km	0.8%
	AB	Single-ended	10.19 km	0.19 km	1.9%
		Double-ended	10.09 km	0.09 km	0.9%
		Double-ended based on Pix2Pix	9.94 km	0.06 km	0.6%
20 km	Ag	Single-ended	20.15 km	0.15 km	0.75%
		Double-ended	20.14 km	0.14 km	0.7%
		Double-ended based on Pix2Pix	20.05 km	0.05 km	0.25%
	AB	Single-ended	19.86 km	0.14 km	0.7%
		Double-ended	20.14 km	0.14 km	0.7%
		Double-ended based on Pix2Pix	20.04 km	0.04 km	0.2%
30 km	Ag	Single-ended	30.22 km	0.22 km	0.73%
		Double-ended	30.22 km	0.22 km	0.73%
		Double-ended based on Pix2Pix	30.15 km	0.15 km	0.5%
	AB	Single-ended	30.30 km	0.30 km	1%
		Double-ended	30.22 km	0.22 km	0.73%
		Double-ended based on Pix2Pix	30.14 km	0.14 km	0.47%
40 km	Ag	Single-ended	40.16 km	0.16 km	0.4%
		Double-ended	40.15 km	0.15 km	0.38%
		Double-ended based on Pix2Pix	40.09 km	0.09 km	0.23%
	AB	Single-ended	40.36 km	0.36 km	0.9%
		Double-ended	40.15 km	0.15 km	0.38%
		Double-Ended based on Pix2Pix	40.01 km	0.01 km	0.03%
50 km	Ag	Single-Ended	51.71 km	1.71 km	3.42%
		Double-Ended	51.08 km	1.08 km	2.16%
		Double-ended based on Pix2Pix	50.88 km	0.88 km	1.76%
	AB	Single-ended	51.56 km	1.56 km	3.12%
		Double-ended	50.93 km	0.93 km	1.86%
		Double-ended based on Pix2Pix	50.67 km	0.67 km	1.35%

## Data Availability

Not applicable.
